# Hemodynamic signal changes and swallowing improvement of repetitive transcranial magnetic stimulation on stroke patients with dysphagia: A randomized controlled study

**DOI:** 10.3389/fneur.2022.918974

**Published:** 2022-08-11

**Authors:** Huiyu Liu, Yang Peng, Zicai Liu, Xin Wen, Fang Li, Lida Zhong, Jinzhu Rao, Li Li, Minghong Wang, Pu Wang

**Affiliations:** ^1^Department of Rehabilitation Medicine, Yue Bei People's Hospital, Shaoguan, China; ^2^School of Rehabilitation Medicine, Gannan Medical University, Ganzhou, China; ^3^Yue Bei People's Hospital, Shaoguan, China; ^4^Department of Rehabilitation Medicine, The 7th Affiliated Hospital of Sun Yat-Sen University, Shenzhen, China

**Keywords:** stroke, dysphagia, functional near-infrared spectroscopy, fNIRS, repeated transcranial magnetic stimulation, rTMS

## Abstract

**Objective:**

Our study aims to measure the cortical correlates of swallowing execution in patients with dysphagia after repetitive transcranial magnetic stimulation (rTMS) therapy using functional near-infrared spectroscopy (fNIRS), and observe the change of pattern of brain activation in stroke patients with dysphagia after rTMS intervention. In addition, we tried to analyze the effect of rTMS on brain activation in dysphagia patients with different lesion sides. This study also concentrated on the effect of stimulating the affected mylohyoid cortical region by 5 Hz rTMS, providing clinical evidence for rTMS therapy of dysphagia in stroke patients.

**Methods:**

This study was a sham-controlled, single-blind, randomized controlled study with a blinded observer. A total of 49 patients completed the study, which was randomized to the rTMS group (*n* = 23) and sham rTMS group (*n* = 26) by the random number table method. The rTMS group received 5 Hz rTMS stimulation to the affected mylohyoid cortical region of the brain and the sham rTMS group underwent rTMS using the same parameters as the rTMS group, except for the position of the coil. Each patient received 2 weeks of stimulation followed by conventional swallowing therapy. Standardized Swallowing Assessment (SSA), Fiberoptic Endoscopic Dysphagia Severity Scale (FEDSS), Penetration-Aspiration Scale (PAS), and functional oral intake status were assessed at two times: baseline (before treatment) and 2 weeks (after intervention). Meanwhile, we use the fNIRS system to measure the cerebral hemodynamic changes during the experimental procedure.

**Results:**

The rTMS group exhibited significant improvement in the SSA scale, FEDSS scale, and PAS scale after rTMS therapy (all *P* < 0.001). The sham rTMS group had the same analysis on the same scales (all *P* < 0.001). There was no significant difference observed in clinical assessments at 2 weeks after baseline between the rTMS group and sham rTMS group (all *P* > 0.05). However, there were statistically significant differences between the two groups in the rate of change in the FEDSS score (*P* = 0.018) and PAS score (*P* = 0.004), except for the SSA score (*P* = 0.067). As for the removal rate of the feeding tube, there was no significant difference between the rTMS group and sham rTMS group (*P* = 0.355), but there was a significant difference compared with the baseline characteristics in both groups (*P*_rTMS_ < 0.001, *P*_shamrTMS_ = 0.002). In fNIRS analysis, the block average result showed differences in brain areas RPFC (right prefrontal cortex) and RMC (right motor cortex) significantly between the rTMS group and sham rTMS group after intervention (*P*_channel30_ = 0.046, *P*_channel16_ = 0.006). In the subgroup analysis, rTMS group was divided into left-rTMS group and right-rTMS group and sham rTMS group was divided into sham left-rTMS group and sham right-rTMS group. The fNIRS results showed no significance in block average and block differential after intervention between the left-rTMS group and sham left-rTMS group, but differences were statistically significant between the right-rTMS group and sham right-rTMS group in block average: channel 30 (T = −2.34, *P* = 0.028) in LPFC (left prefrontal cortex) and 16 (T = 2.54, *P* = 0.018) in RMC. After intervention, there was no significance in left-rTMS group compared with baseline, but in right-rTMS group, channel 27 (T = 2.18, *P* = 0.039) in LPFC and 47 (T = 2.17, *P* = 0.039) in RPFC had significance in block differential. In the sham rTMS group, neither sham left-rTMS group and sham right-rTMS group had significant differences in block average and block differential in each brain area after intervention (*P* > 0.05).

**Conclusions:**

The present study confirmed that a 5-Hz rTMS is feasible at the affected mylohyoid cortical region in post-stroke patients with dysphagia and rTMS therapy can alter cortical excitability. Based on previous studies, there is a dominant hemisphere in swallowing and the results of our fNIRS analysis seemed to show a better increase in cortical activation on the right side than on the left after rTMS of the affected mylohyoid cortical region. However, there was no difference between the left and right hemispheres in the subgroup analysis. Nevertheless, the present study provides a novel and feasible method of applying fNIRS to assessment in stroke patients with dysphagia.

## Introduction

Dysphagia is one of the common complications after stroke, with a prevalence of 37 to 78% (1). The abnormal swallowing process may lead to dehydration, malnutrition, and pneumonia. On the other hand, it prolongs hospital stay, increases medical costs, reduces the quality of life, and even leads to death (2, 3). Therefore, detecting and treating dysphagia as soon as possible is essential for stroke management. For these problems, conventional treatments for dysphagia are taken such as neuromuscular electrical stimulation, feeding training, and adjusting food shapes, as well as intervening eating postures, and so on. Although these treatments are widely applied in clinic, there is no satisfactory effectiveness obtained (4–6).

Recently, the rehabilitation methods of dysphagia are transforming from these compensatory strategies to the retraining of swallowing function based on the principle of neuroplasticity (7). It has been reported that cortical dysfunction can result in swallowing difficulties, and the cerebral cortex plays a crucial role in the initiation and regulation of swallowing (8, 9). According to some previous reviews of the neurophysiology of swallowing, the control of swallowing is more bilaterally implemented in the brain (10, 11). Indeed, some studies suggested that one hemisphere may be dominant for swallowing (12, 13). However, the concept of hemispheric dominance of swallowing resulted from clinical observations of dysphagia patients, which was inferred as a result of neurological examination but not verified radiologically (8, 14, 15). Interestingly, Robbins and Levine suggested that lesions in the left cerebral cortex are associated with impairment of the oral phase of swallowing, whereas lesions in the right hemisphere cortex are associated with impairment of the pharyngeal phase (16). Repetitive transcranial magnetic stimulation (rTMS), which uses magnetic fields to adjust the excitability of the cerebral cortex and speed up neuroplasticity, has been confirmed to improve swallowing function after stroke (17–19). J. W. PARK's study showed that a 5-Hz high-frequency rTMS on the contralesional pharyngeal motor cortex might be helpful for stroke with dysphagia (20). Lee et al. (21) suggested that stimulation of the affected cortex representing the suprahyoid muscle could improve swallowing function. In addition, Vasant et al. (22) indicated that amplitudes of cortical pharyngeal motor evoked potentials (PMEPs) were increased following hemispheric cerebellar rTMS, which was also proved by Lida Zhong's study (23) which showed that cerebellar rTMS is a safe method and represented an effective treatment for dysphagia after stroke. Therefore, it is controversial to determine which stimulation site achieves the better therapeutic and it bases on an understanding of specific areas of the motor cortex involved in swallowing.

Therefore, it is important to assess cortical activity during swallowing to better understand the neural control of swallowing and treat it. There are many clinical evaluation scales for dysphagia, which are subjective and uncertain, because they are mainly based on the clinical measurement and medical history. A relatively recent non-invasive imaging technique, functional near-infrared spectroscopy (fNIRS), measures changes in oxyhemoglobin (oxy-Hb) and deoxyhemoglobin (doxy-Hb) in cortical blood flow. fNIRS provides a safe and non-invasive method to assess cortical activation patterns of patients with neurological disorders. It can provide an important imaging basis for the formulation of clinical rehabilitation programs, and provide real-time feedback on the efficacy of rehabilitation intervention, reflecting and judging the reconstruction of neurological function (24, 25). Several recent studies of fNIRS for swallowing function show that extensive areas of the cerebral cortex are activated in healthy subjects during swallowing tasks (26, 27). Several studies researched that organoleptic testing, such as taste or touch, increases hemodynamic responses in the cortical swallowing network (28, 29). Only one fNIRS study investigated hemodynamic changes in dysphagia patients and healthy controls during the execution of motor imagery and motor imagery of swallowing. The strongest signal changes were observed in the inferior frontal gyrus in both patients and healthy controls (30). In previous studies, results of brain activation patterns of swallowing were mainly based on data from healthy subjects. However, changes in brain activation patterns associated with dysphagia in patients treated with rTMS have been scarcely investigated.

To date, this technique has not been widely applied to examine changes in cortical activation before and after treatment. Thus, we sought to examine the cortical correlates of swallowing in patients with dysphagia after rTMS therapy by using fNIRS, and observe the change of brain activation in stroke patients with dysphagia after rTMS intervention (31). In addition, we tried to analyze the effect of rTMS on brain activation in dysphagia patients with different lesion sides. This study also concentrated on the effect of stimulating the affected mylohyoid cortical region by 5 Hz rTMS, providing clinical evidence for rTMS therapy of dysphagia in stroke patients. Referring to this question, we hypothesized that the swallowing function of patients improved after rTMS therapy and the cortical activation signal enhanced at the affected mylohyoid cortical region where 5 Hz rTMS was stimulated.

## Materials and methods

### Sample size calculation

The patient sample size required for this study was calculated through the website (http://hedwig.mgh.harvard.edu/sample_size/size.html) (32). A previous related study (23) showed that SSA scores (−4.75 ± 4.69) in stroke patients with dysphagia were significantly improved after 5 Hz rTMS therapy. With the statistical power set to 0.9 and at a two-sided 0.05 level of significance, the sample size was calculated to be at least 44, the probability is 90% that the study will detect a treatment difference at a two-sided 0.05 significance level if the true difference between treatments is 4.75 units. This is based on the assumption that the standard deviation of the response variable is 4.69. If 30% of subjects are expected to be lost randomly during the study, a minimum of 57 subjects needs to be recruited.

### Participants

The study was approved by the Ethics Committee of Yue Bei People's Hospital (KY-2021-077, ChiCTR2000032255) and informed consent was obtained from all participants before inclusion. The participants were 60 stroke patients with dysphagia, who were recruited from the Department of Rehabilitation Medicine, Yuebei People's Hospital from July 2020 to January 2021. Besides, each patient was right-handed.

Participants were selected according to the following criteria: (1) stroke patients were diagnosed according to the updated definition for the 21st century (33) and were confirmed to be subcortical stroke by computed tomography (CT) or magnetic resonance imaging (MRI); (2) patients with dysphagia confirmed by fiberoptic endoscopic evaluation of swallowing (FEES); (3) patients with stable vital signs, whose disease duration is between 1 and 12 months and their ages range from 18 to 85 years; and (4) patients without taking the medicine which affects swallowing function. The exclusion criteria included the following: (1) had a history of any other neurogenic diseases, such as epilepsy, tumor, and so on; (2) patients suffering from severe cognitive impairment or aphasia who could not continue to cooperate with treatment; (3) had intracranial metal implants, pacemakers, skull defects, history of seizures, or other contraindications to rTMS treatment; (4) and had a history of sedation, antidepressants, or other drugs that may alter the excitability of the cortex.

### Study design

This study was a sham-controlled, single-blind, randomized controlled study with a blinded observer. A random number table method was used to assign the subjects to a TMS group and a sham TMS group.

A 10-session rTMS was given to the rTMS group for 2 weeks. On the affected mylohyoid cortical region, 5 Hz rTMS was applied. For the sham stimulation group, the same parameters (including position, stimulation frequency, interval time, and pulse number) were used as in the rTMS group. However, to ensure the same stimulation sound without any effective stimulation, the coil was perpendicular to the surface of the skull during the intervention. All patients received the same traditional dysphagia treatment for 30 min each day after rTMS intervention, such as sensory stimulation, tongue retraction exercises, and oropharyngeal muscle strengthening exercises. An experienced physical therapist led the exercises five times each week for 10 days. In all cases, a baseline assessment and a follow-up assessment were carried out at two separate times: baseline and 2 weeks after the intervention.

### Determination of the resting motor threshold (RMT)

Before each patient was treated, RMT was determined. Patients wore positioning caps on their heads and were seated on a comfortable chair with a comfortable position. We request that patients refrain from moving their heads to prevent a change in the site. We then placed a 90-mm figure-eight stimulating coil of MagPro CCY-I stimulator (YIRUIDE Company, Wuhan, China) in the mylohyoid cortical region of the affected hemisphere. The motor evoked potential (MEP) and RMT of each patient were observed by manually controlling the single pulse output. The location of the maximum MEP recording produced in the mylohyoid cortical region is determined as the optional coil position (“hot spot”). The RMT is defined as a stimulus intensity that evoked an MEP >50 UV in at least 5 of 10 successive stimulations across the first dorsal interosseous muscle on the affected side. RMT can be assessed by selecting stimulation locations (mirror regions) at the identical site on the unaffected side if no motor response after maximal stimulation.

### TMS treatment in the control and treatment groups

In the rTMS group, a 5-Hz rTMS protocol was applied to the affected mylohyoid cortical region of the affected hemisphere at an intensity of 80% of RMT, a stimulation time of 2 s, and an interval of 10 s between each stimulation. The treatment duration was 20 min with a total of 100 repetitions and 1,000 stimuli. In the sham rTMS group, the same stimulation parameters were applied. However, to ensure that it generates the same stimulation sound without actually stimulating the scalp, the coil is held at 90° to the scalp during the intervention. For these patients with bilateral brain injury, we selected the side of the brain with more extensive injury or the contralateral brain of a more severely involved limb to determine whether the site of rTMS application was left or right. The treatment period was once a day for 5 days per week for 2 weeks. The rTMS protocols used in our study were based on the safety guidelines for rTMS applications (34).

### Outcome measurements

The primary outcome included the SSA scale and secondary outcomes included assessments of PAS scale, FEDSS scale, and removal rate of the feeding tube. Meanwhile, an fNIRS system is used to detect changes in cerebral hemodynamics during task performance.

### Standardized swallowing assessment (SSA)

SSA is more focused on clinical evidence. It consists of three parts. The first part is the clinical examination, with a total score of 8 to 23, including responsiveness level, and so on. In the second part, patients were given 5 mL of water three times. Its scores vary from 5 to 11 points. What needed to be considered at this time is that if the coughing, choking, breathlessness, or a wet voice were presented by participants. If no problems are evident, 60 mL of water is administered, which is the third part. According to the results, the SSA scores ranged from 18 to 46, with a higher score indicating an impaired swallowing ability (35, 36).

### Fiberoptic endoscopic dysphagia severity scale (FEDSS)

Each participant underwent a fiberoptic endoscopic evaluation of swallowing (FEES) of their swallowing following the clinical assessment. Based on the diverse consistency of diet observed in their endoscopic examination and the risk of saliva penetration or aspiration, stroke-related dysphagia was categorized into a six-point FEDSS scale with 1 score for the best and 6 for the worst (37, 38). Our crew completed the FEES operations, all of whom have several years of experience with this diagnostic instrument.

### Penetration-aspiration scale (PAS)

Each participant in our study was evaluated during FEES by using PAS. The severity of dysphagia was rated by PAS on an eight-point scale. This scale is a semi-quantitative assessment of penetration and aspiration that is commonly used in endoscopic and radiological measurements. The higher the PAS score, the more impaired the swallowing function (39).

### Feeding tube intake status

The functional oral intake status was assessed by using the Functional Oral Intake Scale (FOIS) as feeding tube dependency at assessment (40). There are seven levels in the FOIS scale to describe the functional oral intake of stroke patients with dysphagia. Higher levels indicated better swallowing function (41, 42). Finally, a removal rate was calculated as an outcome.

### Functional near-infrared spectroscopy (fNIRS) data acquisition and swallowing task

**Figure 1:**
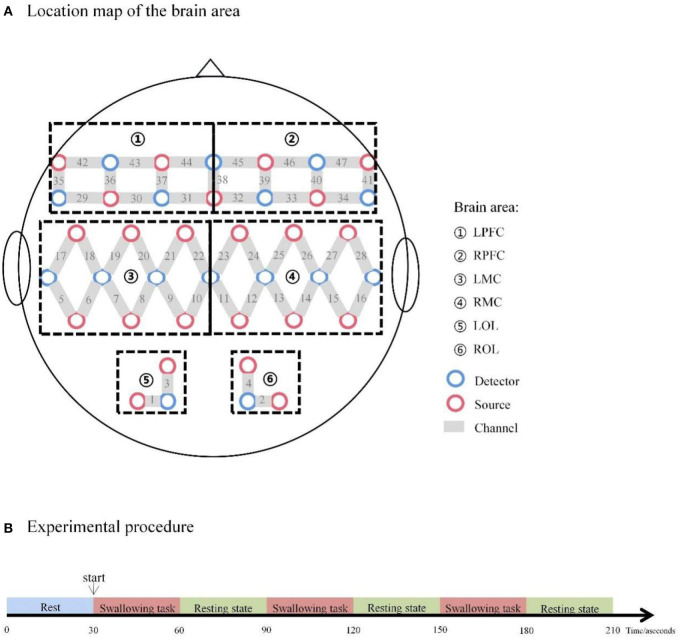
**(A)** Location map of the brain area. **(B)** Experimental procedure. LPFC, left prefrontal cortex; RPFC, right prefrontal cortex; LMC, left motor cortex; RMC, right motor cortex; LOL, left occipital lobe; ROL, right occipital lobe.

fNIRS devices with wavelengths of 730 and 850 nm (NirScan Danyang Huichuang Medical Equipment Co. Ltd., China) were utilized to detect changes in oxyhemoglobin (HbO) and deoxyhemoglobin (HbR) during rest and voluntary swallowing. Multiple regions of the cerebral cortex have been reported as activated areas during swallowing tasks (26, 27). Thus, to identify brain regions and their functions, and to elucidate our study easily, a total of 47 channels were symmetrically positioned over the areas of the prefrontal cortexes (PFCs: LPFC and RPFC), motor cortexes (MCs: LMC and RMC), and occipital lobes (OLs: LOL and ROL) using 23 source optodes and 16 detector optodes with reference to previous studies (43, 44). The NIRS system's sample rate was set to 10 Hz. Figure 1 depicts the channel configuration of the fNIRS probe set. The optodes were placed on the scalp surface of participants according to four reference points (nasion, central zero, left, and right pre-auricular points) based on the anatomical locations defined by the international 10–20 system. The spatial registration method was provided by the NirScan Danyang Huichuang Medical Equipment based on a standard head model setup which used a 3d localization system to confirm the position of the probe falling on the head and further matched into the Broadmann partition. Supplementary Table S1 in the supplemental information showed the brain region and the channel corresponding to the individual Broadmann partition. The participants sited in a quiet fNIRS evaluation room to reduce the influence of noise. To prevent ambient light from entering the system, each optical signal was attached to the skull surface with a custom-made hard plastic cap and covered with a black cloth.

The swallowing procedure used in the present study consisted of two phases of manipulations: 30-s resting-state periods and 30-s swallowing periods. Repeat these two phases three times. The swallowing task required participants to swallow their saliva constantly, as indicated by a voice instruction from the computer. The number of swallows per participant was not controlled but all patients swallowed saliva in the range of 1 to 5 times during the repetitive saliva swallowing test (RSST). To avoid oral movement induced by swallowing movement, each patient rehearsed the swallowing motion before the trial. The whole procedure took approximately 10 min (see Figure 1).

### fNIRS data analysis

The NirSpark package was used to preprocess and analyze the fNIRS data in our study. The following steps were used to preprocess the data: deleting irrelevant time intervals and unrelated artifacts; turning the intensity of light into optical density; choosing the band-pass filter to filter the noise and interference signals (0.01–0.09 Hz); translating optical density to the oxygen concentration in the blood; and setting the initial time of the hemodynamic response function (HRF) to 0 s and the end time to 30 s (the time for a single block paradigm). The blood oxygen concentrations of the three-block paradigms were superimposed and averaged, yielding a block average result with the “swallowing” period set to 30 s. A block differential result was the average of the three-block paradigms of the blood oxygen concentration of swallowing block paradigms minus blood oxygen concentration of rest block paradigms. To analyze the HbO_2_ time-series data, which had been pre-treated for each channel of each subject, and to perform the *t*-test, we utilized a generalized linear model (GLM) (45). The GLM could calculate the degree of matching between the experimental and ideal HRF values for each task and participant (46). The beta value, which represents the channel's level of cortical activation, was utilized to estimate the HRF prediction of the HbO2 signal (47) and can be used to represent the HRF function's peak value. Between-group differences at baseline and after intervention were tested with a two-sample *t*-test in group statistics.

The two sample *t*-test was used to examine between rTMS group and sham rTMS group differences at baseline and the end of the study. Considering that the patient's stimulation sites were divided into left and right sides, which might affect the analysis of fNIRS data, we conducted a subgroup analysis. According to stimulated hemisphere, rTMS group was divided into left-rTMS group and right-rTMS group and sham rTMS group was divided into sham left-rTMS group and sham right-rTMS group. Within-group comparisons of these four groups from baseline to the end of the study were conducted by paired *t*-tests. For stimulation of the left-rTMS group and sham left-rTMS group in the left hemisphere and right-rTMS group and sham right-rTMS group in the right hemisphere, a two-sample *t-*test was used for the analysis of the between-group differences.

### Statistical analysis

All statistical analyses were conducted with SPSS version 26.0 (SPSS Inc). Analyses of exploratory data and Shapiro–Wilk tests were conducted to determine the normality of the data distribution. Continuous variables are presented as mean ± standard deviation. Unpaired *t*-tests or the Mann–Whitney U-test was used to examine between-group differences at baseline and in the change from baseline to the end of the study. Within-group comparisons from baseline to the end of the study used paired *t*-tests or Wilcoxon tests. For categorical variables, counts and percentages are presented. Between-group comparisons at baseline in categorical variables were tested with the χ2 test. A *post-hoc* stimulated hemisphere subgroup analysis was performed to explore the consistency of the results. To control for baseline factors, the rate of change was used in between-group comparisons of the change from baseline to the end of the study of swallowing function assessment. The correlation between HbO_2_ change in each brain area and improvement of SSA in the rTMS group and sham rTMS group were examined using linear regression analysis. A two-sided *P*-value <0.05 was used as the level of significance.

## Results

### Baseline characteristics of the patients

**Figure 2:**
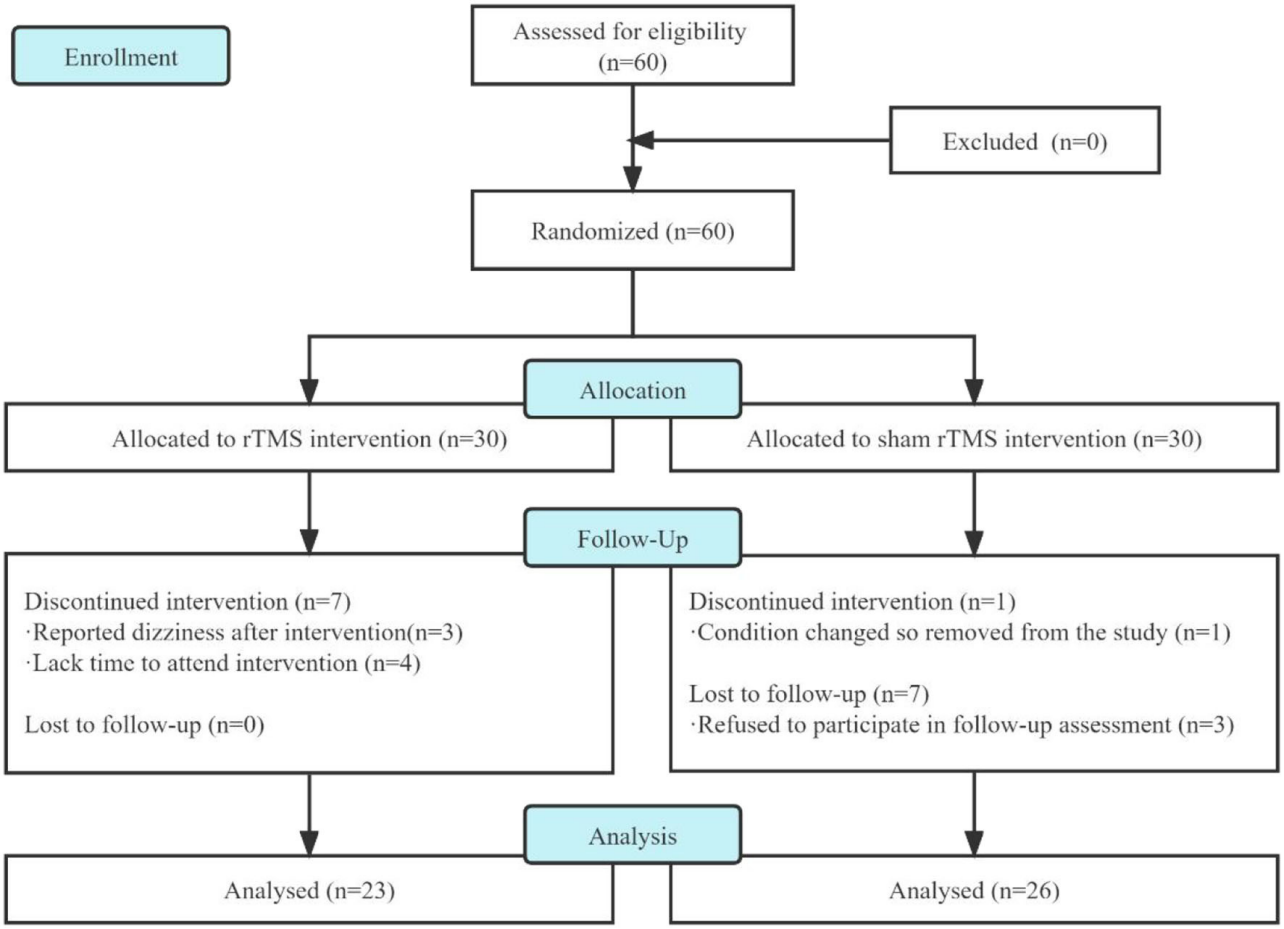
Participant flow diagram.

In this study, we screened 60 patients for eligibility and analyzed only 49 of them who had completed treatment and follow-up assessment (Figure 2). Three patients in the rTMS group dropped out because of dizziness after several interventions, and four patients dropped out of the following intervention due to a lack of time to attend the intervention. In the sham rTMS group, one patient had a change condition so removed from the study and three patients refused to participate in the follow-up assessment.

Table 1 listed the means or medians of the baseline demographic and clinical characteristics of patients. Intergroup comparisons of baseline characteristics revealed no significant differences (*p* > 0.05, see Table 1). For the function assessment at baseline, no significant differences in the scores of MMSE, BADL, WST, SSA, FEDSS, PAS, and number of people with feeding tubes among the two groups were observed (*p* > 0.05, see Table 1).

**Table 1:** Baseline demographic and clinical characteristics.

**Characteristic**	**Mean ± SD**		***P*-value**
	**rTMS group**	**Sham rTMS group**	
Age (years)	67.61 ± 11.71	67.73 ± 9.97	0.969
Sex (M: F)	17: 6	20: 6	0.807
Type of stroke (Hemorrhage: Ischemia)	4: 19	5: 21	0.868
Affected hemisphere (Left: Right: Bilateral)	8: 8: 7	13: 10: 3	0.242
Duration of onset of stroke (days)	74.17 ± 88.20	63.38 ± 59.48	0.888
Hypertension, No. (%)	22 (95.7)	22 (84.6)	0.203
Hyperglycemia, No. (%)	14 (60.9)	10 (38.5)	0.117
MMSE	16.96 ± 8.65	15.12 ± 8.00	0.443
BADL	45.00 ± 26.59	49.42 ± 25.43	0.555
WST	4.17 ± 0.89	4.04 ± 0.82	0.541
SSA	29.35 ± 3.83	27.46 ± 3.35	0.141
FEDSS	4.22 ± 1.00	4.27 ± 1.04	0.909
PAS	4.57 ± 1.31	4.58 ± 1.98	0.759
Feeding tube intake status, No. (%)	12 (52.2)	12 (46.2)	0.674

M, male; F, female; MMSE, Mini-Mental State Examination; BADL, Modified Barthel Index; WST, Water Swallowing Test; SSA, Standardized Swallowing Assessment; FEDSS, Fiberoptic Endoscopic Dysphagia Severity Scale; PAS, Penetration-Aspiration Scale.

### Swallowing function assessments

There was no significant difference observed in the FEDSS score, PAS score, and SSA score at 2 weeks after baseline between the rTMS group and sham rTMS group (all *P* > 0.05, see Table 2). By contrast, in comparison with the baseline characteristics, two groups exhibited significant improvement across all swallowing assessments 2 weeks after baseline (all *P* < 0.001, see Table 2). There was also a significant difference between the rTMS group and sham rTMS group in the rate of change in FEDSS score (*P* = 0.018) and PAS score (*P* = 0.004), except for SSA score (*P* = 0.067, see Table 2).

**Table 2:** The intergroup comparisons of clinical scale scores after the intervention, the intragroup comparisons of clinical scale scores for each group, and intergroup comparisons of the rate of change in clinical scale scores after intervention.

	**Mean** ±**SD**		***P*-value**
	**rTMS group**	**Sham rTMS group**	
**SSA**			
Pre	29.35 ± 3.83	27.46 ± 3.35	0.141
Post	25.57 ± 4.34	24.92 ± 3.88	0.587
The rate of change (%)	−13.09 ± 6.67	−9.33 ± 7.29	0.067
*P*-value	<0.001	<0.001	
**FEDSS**			
Pre	4.22 ± 1.00	4.27 ± 1.04	0.909
Post	2.65 ± 1.11	3.19 ± 0.98	0.073
The rate of change (%)	−38.26 ± 17.40	−25.13 ± 17.17	0.018
*P*-value	<0.001	<0.001	
**PAS**			
Pre	4.57 ± 1.31	4.58 ± 1.98	0.759
Post	2.83 ± 1.47	3.46 ± 1.66	0.105
The rate of change (%)	−39.48 ± 15.95	−23.65 ± 18.57	0.004
*P*-value	<0.001	<0.001	

SSA, Standardized Swallowing Assessment; FEDSS, Fiberoptic Endoscopic Dysphagia Severity Scale; PAS, Penetration-Aspiration Scale.

**Figure 3:**
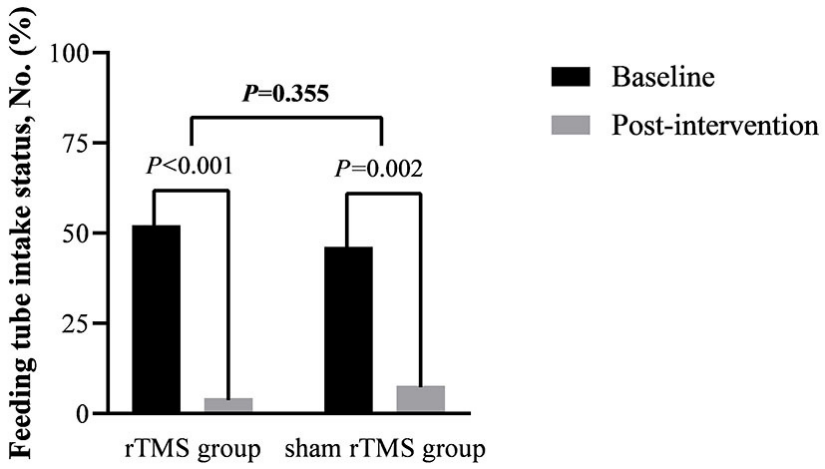
Feeding tube intake status, No. (%).

In the rTMS group, 11 patients pull out the feeding tube successfully after intervention and 10 in the sham rTMS group, which had no significant difference in the removal rate of the feeding tube between groups (*P* = 0.355, see Figure 3). However, there was a significant difference in feeding tube intake status compared with the baseline characteristics in both groups (*P*_rTMS_ < 0.001, *P*_shamrTMS_ = 0.002, see Figure 3).

### Cortical activation analysis of fNIRS measurements

**Figure 4:**
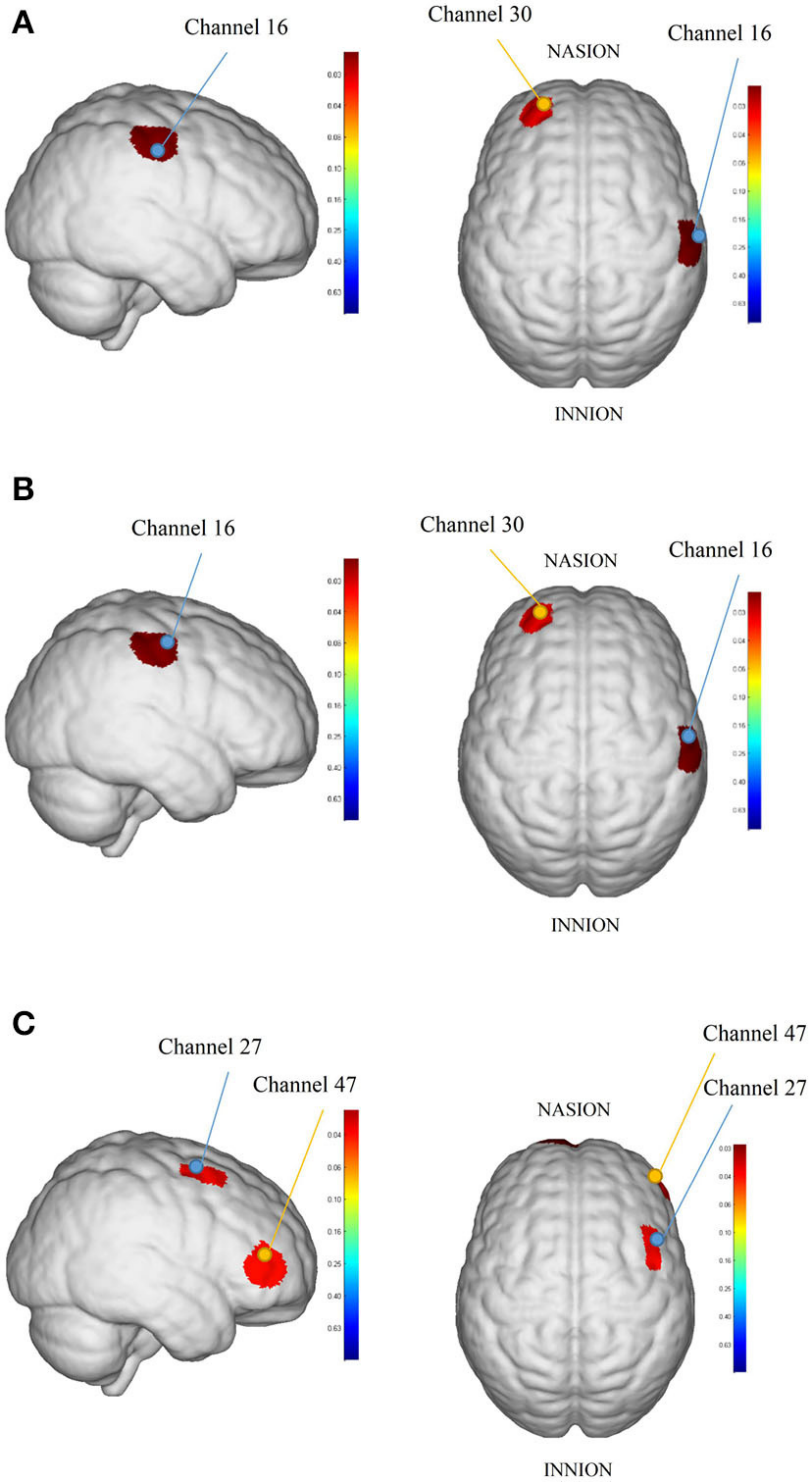
The cortical maps based on analyzed HbO_2_ beta values: **(A)** was the result between rTMS group and sham rTMS group in block average, **(B)** was the result between the right-rTMS group and sham right-rTMS group in block average, and **(C)** was the result between baseline and post-intervention in block differential in the right-rTMS group. The redder color indicates the higher beta value of the channel and the bluer color indicates the lower beta value of the channel.

The HbO_2_ beta values result showed that intergroup comparisons for baseline beta value collection from each brain area between the rTMS group and sham rTMS group demonstrated no significant differences (*P* > 0.05). After the intervention, the block average result showed differences in brain areas RPFC and RMC significantly between the rTMS group and sham rTMS group: channels 30 (T = −2.05, *P* = 0.046) and 16 (T = 2.87, *P* = 0.006). However, there was no significance in block differential between the rTMS group and sham rTMS group after intervention (*P* > 0.05). Beta values of HbO_2_ before and after intervention for each channel in the rTMS group and Sham rTMS group shown in Supplementary Table S2 in Supplemental Information and Figure 4 showed the cortical maps of group analysis.

### Subgroup analysis

We analyzed fNIRS results dividing to left and right which showed that there was no significance in block average and block differential after intervention between the left-rTMS group and sham left-rTMS group. By contrast, the differences in the brain areas RPFC and RMC were statistically significant between the right-rTMS group and sham right-rTMS group in block average: channels 30 (T = −2.34, *P* = 0.028) and 16 (T = 2.54, *P* = 0.018, see Figure 4). After the intervention, there was no significance in HbO_2_ beta collection from each brain area in the left-rTMS group compared with baseline, but in the right-rTMS group, channels 27 (T = 2.18, *P* = 0.039) and 47 (T = 2.17, *P* = 0.039) had significance in block differential (see Figure 4). In the sham rTMS group, neither sham left-rTMS group nor sham right-rTMS group had significant differences in block average and block differential in each brain area after intervention (*P* > 0.05).

**Figure 5:**
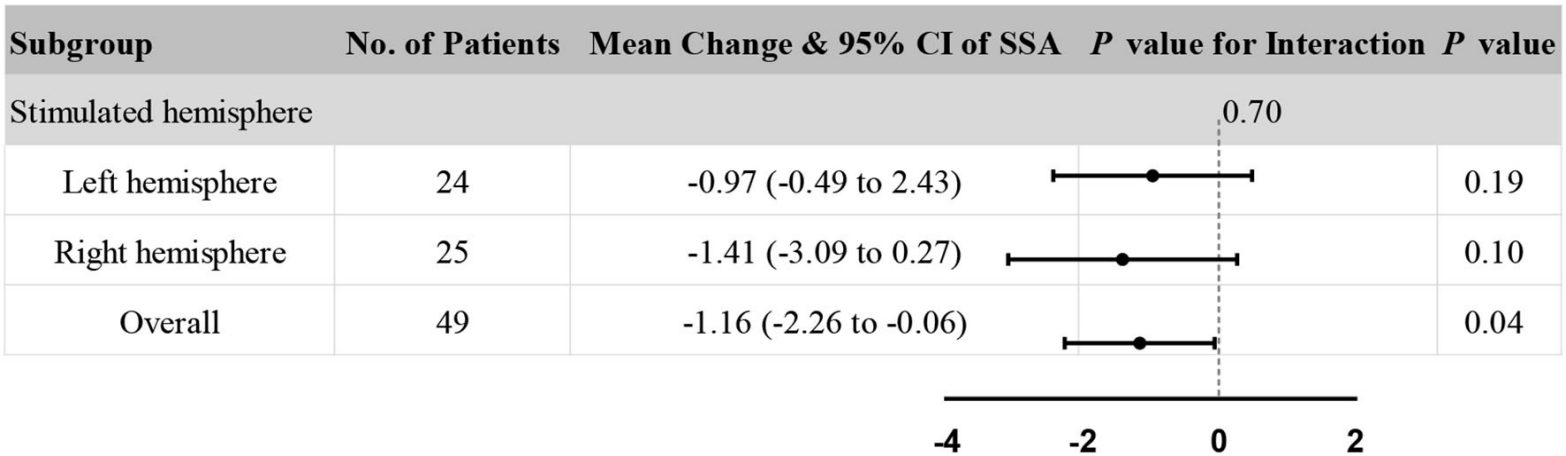
The subgroup analysis of stimulated hemisphere. *P*-value is not adjusted by covariates.

Accordingly, stimulated hemisphere subgroup analysis in clinical assessments was also performed to explore the consistency of the results. The subgroup analysis showed no significant heterogeneity in the stimulated hemisphere (*P* = 0.70 for interaction, see Figure 5). In addition, the rTMS group and sham rTMS group had no significant difference in both the stimulated left hemisphere and the right hemisphere (all *P* > 0.05). Besides, compared to the overall effect (*P* = 0.04), rTMS therapy appeared to have a diminished effect on improving SSA scores, either in the left or right hemisphere.

### The correlation between HbO_2_ change in each brain area and improvement of SSA

The regression equation is not significant in rTMS group (*F* = 2.161, *p* = 0.102) and sham rTMS group (*F* = 0.288, *p* = 0.936) according to the linear regression analysis. The regression analysis yielded an adjusted R square of 0.240 in the rTMS group and −0.206 in the sham rTMS group and all channels' *p*-value of >0.05. Besides, the results of tolerance were <0.2, which indicates that the model could not explain the variance in the SSA improvement.

### Safety and compliance

No serious adverse events or complications were reported during the study. Two patients in the rTMS group reported dizziness after several rTMS interventions. No patients developed seizures during or after therapy.

## Discussion

In the present study, we investigated blood oxygenation level changes in swallowing in stroke patients with dysphagia after rTMS therapy when compared to patients after sham rTMS therapy using fNIRS and the effect of stimulation of the affected mylohyoid cortical region by 5 Hz rTMS. In addition, we tried to analyze the effect of rTMS on brain activation in dysphagia patients with different lesion sides. We found differences in the cortical activation after rTMS therapy between the rTMS group and sham rTMS group.

Compared with the sham rTMS group, the rTMS group showed a significantly more pronounced difference between cortical activation in related swallowing motor areas after the intervention. Furthermore, the rTMS group showed a difference in brain activation after the intervention. Dysphagia patients with left-side rTMS therapy showed no change in cortical activation during swallowing after the intervention. In contrast, dysphagia patients with right-side rTMS therapy showed more right-side cortical activation during swallowing tasks after the intervention. For clinical swallowing function assessments, our results show that poststroke dysphagia improvement could be obtained after a 5-Hz rTMS treatment of the affected hemisphere and traditional dysphagia treatment. The difference in clinical efficacy improvement was more pronounced in the rTMS group compared to the sham rTMS group (*P* < 0.05). Hence, we could show that rTMS applied in the affected mylohyoid cortical region can enhance swallowing function and swallowing motor-related cortical activation in patients with dysphagia after stroke.

### Clinical assessments

Results of the present study demonstrate that rTMS is feasible in stroke patients with dysphagia. This result agrees with that of Liao et al. (48) and Pisegna et al. (49) who reported a positive effect of rTMS on swallowing function recovery after stroke. In general, high-frequency stimulation of the affected hemisphere causes an increase in cortical excitability, while low-frequency stimulation of the unaffected hemisphere causes a decrease in cortical excitability (50). Hummel et al. (51) found that people with brain injuries have significant interhemispheric imbalances, and rTMS stimulation, which modulates cortical excitability, may help minimize this imbalance. In addition, Gow et al. (52) found that 5 Hz rTMS over the swallowing cortex causes an increase in cortex excitability. This study was based on the hypothesis that a high-frequency stimulation to the affected hemisphere increases the excitability of this hemisphere and these changes were likely to lead to increases in cerebral blood flow that ultimately would translate into swallowing improvement.

### The block average results of fNIRS

Importantly, in the present study, we used a multi-channel fNIRS system to monitor cortical blood flow as an evaluation of rTMS intervention. As we expected, the rTMS group showed significant differences compared with the sham rTMS group and the rTMS group showed more activation than the sham stimulation group in related motor areas after rTMS therapy. The main channels where we found differences were channels 16 and 30. Channel 16 was in the RMC based on the international 10–20 system and channel 30 was located in the LPFC.

Previous research has looked into the brain activation patterns associated with active swallowing. Kyoko's study (27) found extensive cortical activation in 15 healthy subjects during volitional swallowing using multichannel fNIRS, including bilateral anterior central gyrus, anterior central gyrus, inferior frontal gyrus, superior temporal gyrus, superior temporal gyrus, and superior temporal gyrus, which was consistent with the results of Kern's study (8) using fMRI. Among them, an extensive cortical activation in healthy subjects was the same as the result of Martin's study (53), but the activated signal of the inferior frontal gyrus was the strongest. Xurui Liu's study (54) demonstrated that the oxyhemoglobin (HbO_2_) concentration was decreased during stimulation, and then raised during different stimulation frequencies. The result is the same as our results which showed that the prefrontal lobe may be involved in swallowing and rTMS therapy can alter cortical excitability.

### The block differential results of fNIRS

Previous studies found that oxy-Hb peaking occurred ~15 s after task initiation in healthy subjects (30). However, most patients with dysphagia may take as much as 4 to 6 s to complete the task, unlike healthy individuals who can swallow within 1 s. Besides, as a result of the stroke, blood flow will initially be affected in the lesioned hemisphere which may affect measurements of the hemodynamic response. Therefore, we compared the difference which is determined as the difference in patients after stroke resting state and swallowing task. The results of our study showed that there was no change in block differential results after dysphagia patients with left-side rTMS therapy. On the contrary, dysphagia patients with right-side rTMS therapy showed more differences in right-side cortical activation after the intervention. In addition, there were differences in activation of the right hemisphere between the rTMS group and sham rTMS group.

This may be related to cortical activation patterns in the process of swallowing. Prior studies report that there were mainly bilateral patterns of cortical activation during swallowing and no lateralization was found (26). However, recent studies tend to suggest that one or the other hemisphere may be dominant in humans after brain damage. Hamdy's study (55) reported that multiple cerebral regions are recruited asymmetrically during swallowing, particularly in the right insula and left cerebellum. Jiahui Tai et al. (56) found significant hemispheric asymmetry in brain activation by using fNIRS to detect 22 healthy adults performing a swallowing task. It has also been shown that hemispheric dominance appeared to be different in some subjects depending on the swallowing task (57). For patients, Gallas et al. (58) evaluated mylohyoid motor evoked potentials (MHMEPs) in stroke patients with aspiration, or residue or without swallowing problem, determining a dominant hemisphere and providing evidence for asymmetry in the mylohyoid cortical region of the brain. Kober et al. (30) found that dysphagia patients with right hemisphere lesions are more likely to demonstrate unilateral patterns of activity during swallowing, which our fNIRS results are consistent with it.

However, the subgroup analysis of the stimulated hemisphere showed that there was no significant heterogeneity in the stimulated hemisphere in SSA scores (*P* = 0.70 for interaction, see Figure 3). The result was similar to a previous review which reported that no significant differences were found dependent on the stimulation site (59). The bilateral cerebral hemispheres may play a role in swallowing, so stimulating either hemisphere may promote swallowing function.

Many previous studies based on fMRI, magnetoencephalography (MEG), and fNIRS provide evidence that multiple cerebral regions were activated during swallowing in healthy participants (26, 60, 61). As for rTMS therapy in dysphagia, previous reviews have found that rTMS can improve swallowing function, whether stimulating the healthy hemisphere, the affected hemisphere, or both hemispheres (48, 49). Although the rTMS site in our study was on the affected hemisphere, a significant difference in block average of both channel 30 and channel 16 was found in the fNIRS of the rTMS group after the intervention, in contrast to the sham rTMS group. Channel 30 was located in the left prefrontal cortex and channel 16 was located in the right primary motor cortex and pre-motor and supplementary motor cortex. If the changes in channels 30 and 16 observed in our study are due to rTMS, we can speculate that high-frequency rTMS stimulation enhances cortical activation. A recent study found a significant increase in oxy- and deoxy-Hb during swallowing in the affected hemisphere in stroke patients with right hemisphere lesions. In contrast, the healthy hemisphere was less active during swallowing (30). However, only two cases of patients were reported in this study, and further studies with a large sample are still needed. It has been demonstrated that high-frequency rTMS increased cortical excitability in the stimulated affected hemisphere which enhanced motor function by increases in cerebral blood flow (62, 63). Besides, an increase in cerebral activation in the unaffected hemisphere is connected to a natural recovery of swallowing following a stroke. For instance, Handy et al. (64) researched the projections from both hemispheres to the swallowing muscles in a large group of patients with pure unilateral stroke and they discovered that patients with dysphagia have smaller pharyngeal responses on the unaffected hemisphere than those who can swallow normally. Therefore, it is possible that the bilateral cerebral hemispheres play a role in swallowing and may relate to interhemispheric differences in brain morphology and influences the effects of rTMS. This could explain why rTMS have effects on different cortical areas because it could regulate cerebral blood flow to reorganize neuronal networks.

Considering that the intact hemisphere plays a vital role in the recovery of swallowing after stroke, we have a fascinating opportunity to study the plasticity of a normal path. Previously it has been shown that Giovanni et al. (65) proposed a bimodal balance–recovery model which connects interhemispheric balancing and functional recovery to the structural reserve spared by the lesion. One underlying mechanism for such a model might be a choice of interhemispheric competition model and vicariation model of motor recovery after stroke which leads to the best NIBS interventions tailored to the needs of individual patients. According to our research, it could be suggested that future high-frequency rTMS therapies to enhance swallowing function recovery after stroke may attempt toward manipulating reorganization on the affected side.

In the present study, the HbO_2_ change in each brain area was not associated with improvement of SSA, potentially because of the small sample size. With limited sample size, we did not conduct the linear regression analysis with the change in HbO_2_ at each brain area instead of the change in HbO_2_ at each channel as the explanatory variable. In addition, we thought that the selection of explanatory variables is complex due to the extensive channel distribution. Therefore, larger sample size is needed for further correlation studies.

Although very preliminary, the application of NIBS and non-invasive neuroimaging techniques is becoming more widespread, which can be used to either act on or monitor cortical regions. LTP, LTD, changes in cerebral blood flow, the activity of specific enzymes, relationships between cortical and subcortical structures, and gene expression may be influenced during the NIBS therapy and benefits standard therapy (66). The fNIRS and other non-invasive neuroimaging techniques can be also used to study abnormal cortical activation during swallowing and other actions in patients with brain injury or neurological disease. Once through the repeated use of fNIRS or other non-invasive neuroimaging techniques in patients as was recently done in swallowing, then we can understand the patient's brain mechanisms fully that choose the best NIBS interventions tailored to the needs of individual patients. Researchers or therapists can envisage how these techniques might be applied during the traditional therapy process as a way of improving their effectiveness. However, a large number of studies using neuroimaging and neuromodulation in clinical research with patients are required before this strategy to test this hypothesis and test it in practice.

### Limitations

There are some limitations to this study. First, patients with ischemic and hemorrhagic strokes, oral phase swallowing disorder, and pharyngeal phase swallowing disorder were grouped together because of the small sample size, even though these factors could affect recovery. It is unfortunate that recent trials have been unable to overcome this drawback. Second, the stimulation coil was hand-fixed because of lacking of sham coil, which is not the most adequate blinding. Third, we did not control the number of saliva swallows for each patient and did not record the specific saliva swallow times for each patient during fNIRS, which may influence the changes in HbO_2_. Finally, the application of different fNIRS parameters as feedback signals, such as oxy-Hb, deoxy-Hb, beta values, and slope values, would be a more valuable experiment. This would allow us to test if the different parameters can lead to different results. Hence, further studies have to process these subgroups and with larger samples to demonstrate the potential benefits of fNIRS and NIBS intervention of swallowing for dysphagia patients and use more accurate experimental equipment to reduce experimental error and bias.

## Conclusion

The present study confirmed that a 5-Hz rTMS is feasible at the affected mylohyoid cortical region in post-stroke patients with dysphagia and rTMS therapy can alter cortical excitability. Based on previous studies, there is a dominant hemisphere in swallowing and the results of our fNIRS analysis seemed to show a better increase in cortical activation on the right side than on the left after rTMS of the affected mylohyoid cortical region. However, there was no difference between the left and right hemisphere in the subgroup analysis. Nevertheless, present study provides a novel and feasible method of applying fNIRS to assessment in stroke patients with dysphagia.

## Data availability statement

The raw data supporting the conclusions of this article will be made available by the authors, without undue reservation.

## Ethics statement

The studies involving human participants were reviewed and approved by the Ethics Committee of Yue Bei People's Hospital. The patients/participants provided their written informed consent to participate in this study.

## Author contributions

LL, PW, and MW were involved in the conception of the trial and supervised the study. HL and YP performed data sorting and analysis, and manuscript writing and editing. ZL, XW, and FL performed data collection. LZ and JR provided suggestions for data analysis. All authors contributed to the article and approved the submitted version.

## Conflict of interest

The authors declare that the research was conducted in the absence of any commercial or financial relationships that could be construed as a potential conflict of interest.

## Publisher's note

All claims expressed in this article are solely those of the authors and do not necessarily represent those of their affiliated organizations, or those of the publisher, the editors and the reviewers. Any product that may be evaluated in this article, or claim that may be made by its manufacturer, is not guaranteed or endorsed by the publisher.
