# Supplementary material for: Hemodynamic signal changes and swallowing improvement of repetitive transcranial magnetic stimulation on stroke patients with dysphagia: A randomized controlled study
Source: Front Neurol. 2022 Aug 11;13:918974. doi: 10.3389/fneur.2022.918974 (PMC9403609; doi:10.3389/fneur.2022.918974)
Supplement: Supplementary file 1 [file Data_Sheet_1.docx]

**Supplemental Information**

| **Brain area** | **Channel** | **Broadmann partition** |
| --- | --- | --- |
| LPFC | 29,30,31,35,36,37,42,43,44 | 9 - Dorsolateral prefrontal cortex  10 - Frontopolar area 45 - pars triangularis Broca’s area 46 - Dorsolateral prefrontal cortex 47 - Inferior prefrontal gyrus |
| RPFC | 32,33,34,38,39,40,41,45,46,47 | 9 - Dorsolateral prefrontal cortex  10 - Frontopolar area 44 - pars opercularis part of Broca’s area  45 - pars triangularis Broca’s area 46 - Dorsolateral prefrontal cortex 47 - Inferior prefrontal gyrus |
| LMC | 5,6,7,8,9,10,17,18,19,20,21,22 | 1 - Primary Somatosensory Cortex 2 - Primary Somatosensory Cortex  3 - Primary Somatosensory Cortex 4 - Primary Motor Cortex 6 - Pre-Motor and Supplementary Motor Cortex 8 - Includes Frontal eye fields 9 - Dorsolateral prefrontal cortex 40 - Supramarginal gyrus part of Wernicke’s area |
| RMC | 11,12,13,14,15,16,23,24,25,26,27,28 | 1 - Primary Somatosensory Cortex 2 - Primary Somatosensory Cortex  3 - Primary Somatosensory Cortex 4 - Primary Motor Cortex  6 - Pre-Motor and Supplementary Motor Cortex 8 - Includes Frontal eye fields 9 - Dorsolateral prefrontal cortex  40 - Supramarginal gyrus part of Wernicke’s area |
| LOL | 1,3 | 18 - Visual Association Cortex (V2) 19 - Visual Association Cortex (V3) |
| ROL | 2,4 | 18 - Visual Association Cortex (V2) 19 - Visual Association Cortex (V3) |

Table S1 brain region and channel corresponding to the individual Broadmann partition. LPFC: left prefrontal cortex; RPFC: right prefrontal cortex; LMC: left motor cortex; RMC: right motor cortex; LOL: left occipital lobe; ROL: right occipital lobe.

| **Channel** | **rTMS group** | | **Sham rTMS group** | |
| --- | --- | --- | --- | --- |
|  | Baseline | Post-intervention | Baseline | Post-intervention |
| 1 | 0.0357±0.06706 | -0.0034±0.07920 | -0.0020±0.08188 | 0.0159±0.05871 |
| 2 | 0.0139±0.12020 | 0.0149±0.06370 | -0.0089±0.05299 | -0.0078±0.06739 |
| 3 | 0.0065±0.08291 | 0.0154±0.05198 | 0.0076±0.07683 | 0.0023±0.08272 |
| 4 | 0.0024±0.04514 | -0.0015±0.06703 | 0.0101±0.05860 | -0.0083±0.07582 |
| 5 | 0.0262±0.03815 | 0.0225±0.04382 | 0.0085±0.03938 | 0.0224±0.06816 |
| 6 | 0.0220±0.03334 | 0.0244±0.05169 | 0.0064±0.02833 | 0.0100±0.03470 |
| 7 | 0.0256±0.03903 | 0.0192±0.04771 | 0.0113±0.03139 | 0.0085±0.03299 |
| 8 | 0.0224±0.03050 | 0.0079±0.04662 | 0.0075±0.03914 | 0.0007±0.02499 |
| 9 | 0.0090±0.04358 | 0.0219±0.04251 | -0.0097±0.07884 | 0.0017±0.04399 |
| 10 | 0.0177±0.03559 | 0.0169±0.02611 | -0.0042±0.03806 | -0.0051±0.03737 |
| 11 | 0.0194±0.03104 | 0.0013±0.07383 | -0.0059±0.03008 | 0.0103±0.04845 |
| 12 | 0.0350±0.06778 | -0.0113±0.13084 | 0.0077±0.02860 | -0.0150±0.05842 |
| 13 | 0.0151±0.04557 | 0.0097±0.02800 | 0.0070±0.03668 | -0.0009±0.04600 |
| 14 | 0.0087±0.04847 | 0.0015±0.08112 | 0.0085±0.03512 | 0.0119±0.04244 |
| 15 | 0.0077±0.04581 | 0.0121±0.04437 | -0.0071±0.03215 | 0.0042±0.02416 |
| 16 | 0.0235±0.04214 | 0.0148±0.04446 | 0.0037±0.03014 | 0.0241±0.05286 |
| 17 | 0.0371±0.03948 | 0.0063±0.04488 | 0.0056±0.05001 | 0.0104±0.05889 |
| 18 | 0.0370±0.04193 | 0.0142±0.05257 | 0.0098±0.04212 | 0.0143±0.05312 |
| 19 | 0.0108±0.04488 | 0.0117±0.05513 | 0.0154±0.02764 | 0.0031±0.02552 |
| 20 | 0.0143±0.03201 | 0.0137±0.04166 | 0.0090±0.03542 | 0.0021±0.02583 |
| 21 | 0.0091±0.05706 | 0.0055±0.04463 | -0.0019±0.04499 | 0.0043±0.04607 |
| 22 | 0.0107±0.03904 | -0.0019±0.03499 | -0.0004±0.04583 | 0.0099±0.03900 |
| 23 | 0.0141±0.03596 | 0.0086±0.02839 | 0.0040±0.02654 | 0.0039±0.02863 |
| 24 | 0.0095±0.04701 | 0.0409±0.07474 | 0.0066±0.04344 | -0.0019±0.04260 |
| 25 | 0.0203±0.02886 | 0.0079±0.03210 | 0.0030±0.02066 | 0.0049±0.03213 |
| 26 | 0.0231±0.04487 | 0.0147±0.02980 | -0.0010±0.03285 | 0.0062±0.03530 |
| 27 | 0.0274±0.03788 | 0.0266±0.05606 | 0.0033±0.05739 | 0.0144±0.05389 |
| 28 | 0.0344±0.04831 | 0.0212±0.03743 | 0.0003±0.03843 | 0.0010±0.03284 |
| 29 | 0.0426±0.05555 | 0.0192±0.03962 | 0.0124±0.03561 | -0.0002±0.06438 |
| 30 | 0.0294±0.05240 | 0.0237±0.05331 | 0.0141±0.05667 | -0.0062±0.03709 |
| 31 | 0.0168±0.04595 | 0.0231±0.05527 | 0.0035±0.03890 | 0.0134±0.05177 |
| 32 | 0.0152±0.04026 | 0.0157±0.05113 | -0.0048±0.04654 | 0.0010±0.03908 |
| 33 | 0.0194±0.04426 | 0.0167±0.02968 | 0.0046±0.05018 | 0.0048±0.04843 |
| 34 | 0.0294±0.04978 | 0.0019±0.08524 | 0.0101±0.04695 | 0.0161±0.03060 |
| 35 | 0.0374±0.05580 | 0.0238±0.07877 | 0.0344±0.06878 | 0.0241±0.06883 |
| 36 | 0.0188±0.05743 | 0.0307±0.06714 | 0.0161±0.04942 | 0.0014±0.05028 |
| 37 | 0.0431±0.08800 | 0.0083±0.05342 | 0.0177±0.03797 | -0.0060±0.06368 |
| 38 | 0.0024±0.05894 | 0.0073±0.03674 | -0.0027±0.03302 | -0.0023±0.04315 |
| 39 | 0.0347±0.05653 | 0.0094±0.04975 | -0.0055±0.04646 | 0.0000±0.05090 |
| 40 | 0.0377±0.06875 | 0.0149±0.04997 | 0.0274±0.06480 | 0.0075±0.053333 |
| 41 | 0.0199±0.06489 | 0.0400±0.09178 | 0.0315±0.05952 | 0.0289±0.07456 |
| 42 | 0.0403±0.09640 | 0.0273±0.06593 | 0.0212±0.04562 | 0.0159±0.05019 |
| 43 | 0.0265±0.07425 | 0.0103±0.05095 | 0.0155±0.05159 | -0.0041±0.04764 |
| 44 | 0.0265±0.06848 | 0.0219±0..05628 | 0.0066±0.02247 | 0.0016±0.03458 |
| 45 | 0.0155±0.06893 | 0.0010±0.04366 | 0.0028±0.03209 | -0.0019±0.03338 |
| 46 | 0.0222±0.07357 | 0.0103±0.07741 | -0.0066±0.04883 | -0.0006±0.05098 |
| 47 | 0.0578±0.07448 | 0.0348±0.09055 | 0.0219±0.05410 | 0.0305±0.09573 |

Table S2 Beta values of HbO_2_ before and after intervention for each channel in rTMS group and Sham rTMS group.
